# The Lack of CuZnSOD Leads to Impaired Neurotransmitter Release, Neuromuscular Junction Destabilization and Reduced Muscle Strength in Mice

**DOI:** 10.1371/journal.pone.0100834

**Published:** 2014-06-27

**Authors:** Yun Shi, Maxim V. Ivannikov, Michael E. Walsh, Yuhong Liu, Yiqiang Zhang, Carlos A. Jaramillo, Gregory T. Macleod, Holly Van Remmen

**Affiliations:** 1 Barshop Institute for Longevity and Aging Studies, University of Texas Health Science Center at San Antonio, San Antonio, Texas, United States of America; 2 Department of Cellular and Structural Biology, University of Texas Health Science Center at San Antonio, San Antonio, Texas, United States of America; 3 Geriatric Research Education and Clinical Center, South Texas Veterans Health Care System, San Antonio, Texas, United States of America; 4 Department of Physiology, University of Texas Health Science Center at San Antonio, San Antonio, Texas, United States of America; 5 Department of Rehabilitation Medicine, University of Texas Health Science Center at San Antonio, San Antonio, Texas, United States of America; 6 Oklahoma City VA Medical Center, Oklahoma City, Oklahoma, United States of America; University of Sydney, Australia

## Abstract

Elevated reactive oxygen species (ROS) production and ROS-dependent protein damage is a common observation in the pathogenesis of many muscle wasting disorders, including sarcopenia. However, the contribution of elevated ROS levels to –a breakdown in neuromuscular communication and muscle atrophy remains unknown. In this study, we examined a copper zinc superoxide dismutase [CuZnSOD (Sod1)] knockout mouse (*Sod1* ^−/−^), a mouse model of elevated oxidative stress that exhibits accelerated loss of muscle mass, which recapitulates many phenotypes of sarcopenia as early as 5 months of age. We found that young adult *Sod1* ^−/−^ mice display a considerable reduction in hind limb skeletal muscle mass and strength when compared to age-matched wild-type mice. These changes are accompanied by gross alterations in neuromuscular junction (NMJ) morphology, including reduced occupancy of the motor endplates by axons, terminal sprouting and axon thinning and irregular swelling. Surprisingly however, the average density of acetylcholine receptors in endplates is preserved. Using *in vivo* electromyography and *ex vivo* electrophysiological studies of hind limb muscles in *Sod1* ^−/−^ mice, we found that motor axons innervating the extensor digitorum longus (EDL) and gastrocnemius muscles release fewer synaptic vesicles upon nerve stimulation. Recordings from individually identified EDL NMJs show that reductions in neurotransmitter release are apparent in the *Sod1* ^−/−^ mice even when endplates are close to fully innervated. However, electrophysiological properties, such as input resistance, resting membrane potential and spontaneous neurotransmitter release kinetics (but not frequency) are similar between EDL muscles of *Sod1* ^−/−^ and wild-type mice. Administration of the potassium channel blocker 3,4-diaminopyridine, which broadens the presynaptic action potential, improves both neurotransmitter release and muscle strength. Together, these results suggest that ROS-associated motor nerve terminal dysfunction is a contributor to the observed muscle changes in *Sod1* ^−/−^ mice.

## Introduction

There is a delicate balance between reactive oxygen species (ROS) production and detoxification resulting in a limited amount of free ROS. At low levels, ROS have been shown to exert a modulatory action on a number of intracellular signaling pathways and ion channels [1]–[3]. However, in certain conditions such as advanced age and in many neurodegenerative disorders, there is a substantial increase in ROS production that can overwhelm antioxidant mechanisms and give rise to higher levels of unscavenged ROS [4], [5]. The resultant oxidative stress is believed to be responsible for many tissue changes including the loss of skeletal muscle mass and strength, impairment of neurotransmitter release, and neuronal degeneration.

Experimentally reducing cellular antioxidant capacity by disruption of the Cu/Zn superoxide dismutase gene in mice (*Sod1* ^−/−^) results in very high levels of oxidative stress and oxidative damage in all tissues and an acceleration of age-related loss of skeletal muscle mass [6], [7]. Skeletal muscle atrophy in these mice is accompanied by neuromuscular junction (NMJ) morphologic changes, increased denervation and an elevated production of superoxide and hydrogen peroxide by muscle mitochondria [6], [8]. The loss of CuZnSOD in muscle is also associated with significant muscle weakness [7]. Elevated levels of ROS can contribute to muscle loss and weakness through a variety of pathways, e.g., oxidative damage and elevated degradation of contractile proteins or oxidative modification of proteins involved in calcium homeostasis and excitation contraction coupling. Indeed, elevated ROS have been shown to activate calpain and ubiquitin proteolytic systems and could thus lead to loss of muscle mass [9], [10].

Oxidative stress may also contribute to muscle atrophy and weakness via its effects on neurotransmission at NMJs. For example, elevated synaptic ROS levels have been shown to decrease glutamate reuptake after release in rat brain synaptosomes [11]. Transient application of hydrogen peroxide and Fe^2+^ to frog and mouse NMJs inhibits both spontaneous and evoked release [12]. These inhibitory effects of exogenous ROS on spontaneous release closely resemble alterations in neurotransmission seen in mouse models of amyotrophic lateral sclerosis that overexpress mutant variants of human SOD1 [5]. On the other hand, direct inhibition of neurotransmitter release at NMJs with botulinum toxin A or tetrodotoxin injections in mice produces marked skeletal muscle atrophy [13], [14]. However, the impact of elevated oxidative stress on neurotransmission and its downstream effects on muscle atrophy or muscle weakness *in vivo* are not well defined.

In this study, we utilized *Sod1* ^−/−^ mice to probe the role of oxidative stress in neurotransmitter release and synaptic transmission. We show that adult *Sod1* ^−/−^ mice (∼ 7 month old) display hind limb atrophy and weakness, and reduced compound muscle action potential (CMAP) parameters when compared to age-matched wild-type controls. Consistent with reduced CMAP measurements, electrophysiological recordings from individual muscle fibers reveal a substantial decrease in neurotransmitter release. Morphological analyses of NMJs performed together with the electrophysiological recordings suggest that changes in synaptic release likely precede NMJ denervation. In agreement with a presynaptic deficit hypothesis, administration of 3,4-diaminopyridine, a potassium channel blocker which increases action potential duration, improves synaptic transmission and contributes to an increase in muscle strength.

## Materials and Methods

### Ethics statement

All experiments were performed in accordance with the National Institutes of Health *Guide for the Care and Use of Laboratory Animals*. Experimental protocols were approved by the Institutional Animal Care and Use Committee at the University of Texas Health Science Center at San Antonio (IACUC # 08080z) and the Audie L. Murphy VA Hospital.

### Mice

CuZnSOD deficient mice (*Sod1* ^−/−^) were originally derived in Dr. Charles Epstein's laboratory at the University of California, San Francisco [15]. They are maintained in C57Bl/6 background. Thy1-YFP (line 16) mice were purchased from the Jackson Laboratory and crossed to *Sod1* ^−/−^ mice to generate *Thy1-YFP* ^tg/+^::*Sod1* ^+/+^and *Thy1-YFP* ^tg/+^::*Sod1* ^−/−^ mice for NMJ imaging experiments. Mice were fed a standard rodent chow diet *ad libitum* and kept in a temperature and humidity controlled environment with artificial lighting (12h dark/12h light). All experiments were performed using young adult animals aged between 4 and 10 months old. The average age of mice used for each experiment is noted in the figure legends. Tail clips were used for DNA isolation and genotyping PCR.

### Grip strength and wire hanging test

*Sod1* ^−/−^ mice have previously been shown to have a significant reduction in hind limb contractile function measured *in situ*, even in young adult mice at 8 months of age [7]. In this study, we measured fore- and hind-limb grip strength using a Grip Strength Meter with mesh grid pull bar (Columbus Instruments 1027 CSM) specifically designed for mice. Mice were allowed to grasp the pull bar with both fore limbs and hind limbs, and were then gradually pulled backward in a horizontal plane until they lost their grip. Mice were not trained prior to testing. The average value of ten consecutive measurements was designated as the mouse's grip strength. Grip strength was also tested 10 minutes after injection with 3,4-diaminopyridine (DAP;1 mg/kg in saline, i.p.) or saline (control) to evaluate the effect of neurotransmission potentiation on muscle strength. Preliminary studies using high dose of DAP (8 mg/kg) as shown in the electromyography studies had caused occasional involuntary muscle twitching, therefore a lower dose was used to consistently evaluate the effect on grip strength. No adverse effects were observed at 1 mg/kg.

The wire hanging test was performed to evaluate motor function and neuromuscular grip strength [16], [17]. Briefly, it was determined by positioning individual mouse on a cross-grip wire rack and then the rack was inverted and the latency to fall was recorded for each trial. The average latency to fall of four to five trials was calculated for each animal.

### In vivo electrodiagnostic studies

The animals were anesthetized with isoflurane as described previously [18] and maintained at 33–34°C under a heating lamp. All experiments were performed with a portable Nicolet Viking Quest portable EMG apparatus (CareFusion, San Diego, CA, USA). Nerve conduction velocity experiments include sciatic nerve conduction velocity (NCV), sural NCV and tail distal motor latency (TDML) tests were performed as described previously [18]. Among these tests, sciatic NCV and TDML specifically evaluate motor nerve function, while sural NCV involves sensory nerve only. TDML test is performed by stimulating tail motor nerve and measuring the latency between stimulation and the initial onset of compound muscle action potential (CMAP) recorded 30 mm distal to the stimulating electrode. It reflects both motor nerve conduction velocity and neurotransmission across NMJs at these nerve terminals. The repetitive nerve stimulation (RNS) method was adopted from Kaja et al. (2007) with minor modifications [19]. The stimulating electrode was inserted near the sciatic nerve in the thigh. The reference electrode was placed subcutaneously about 1 cm away from the stimulation needle electrode. The evoked CMAPs from the gastrocnemius were recorded with a subcutaneous electrode that was placed over the mid-belly of the sampling muscle near the endplate zone. The reference electrode was placed at the ankle and a ground electrode was placed at the tail. Electrical stimuli of supramaximal intensity were delivered to the sciatic nerve to ensure activation of all the motor axons innervating the muscle. The extracellular recorded CMAP has a large initial biphasic waveform as a result of propagation of the action potential away from the recording electrode at the endplate zone. The delayed smaller peaks towards the end of each trace represent the F-wave. It is a result of an antidromic motor response to the spinal cord followed by an orthrodromic motor response back to the recording electrodes. Trains of 10 stimuli were applied at various frequencies (0.2, 3, 5 and 10 Hz) with a 2-min recovery period between successive trains. CMAP amplitude and area of baseline to the initial negative peak were measured for all CMAPs in a train. Representative traces of CMAP at 0.2 and 10 Hz are shown with 1ms interval for easy visualization. By comparing the amplitude of the fifth evoked CMAP with the first one, the decrease percentage was reported as decrement. The effect of 3,4-diaminopyridine on neurotransmission was examined by i.p. injection of DAP solution (8 mg/kg in saline) and conducting RNS test 20 minutes later. The CMAP decrement was compared to values obtained prior to the injection.

### Intracellular recording at NMJs

Mice were euthanized and either the left or right hind limb was shaved and skinned. After removal of the biceps femoris muscle and sciatic nerve dissection, a complex of the EDL and tibialis anterior (TA) muscles was dissected out and pinned by the tendons into a 35 mm Sylgard coated dishes containing oxygenated perfused Ringer's solution. For electrical stimulation of the NMJ, the common peroneal nerve was drawn into a glass suction electrode and stimulated at the desired frequency. µ-conotoxin GIIIB (2 µM, Peptide International, Louisville, Kentucky, USA) was used to block muscle contractions. Electrophysiological recordings of individual muscle fibers were made using a 10X water-immersion objective of a BX51WI Olympus microscope. Micropipettes were filled with a mixture of KCl and K-acetate solution. A 1∶1 mixture of 3 M KCl and K-acetate solutions was used because chloride ions leak from the pipette into the muscle, making the resting membrane potential recordings unstable. Inclusion of an additional ion other than chloride (acetate/citrate) improves the stability of recordings. To make the recordings, each electrode was connected to an Axoclamp 900A amplifier through 0.1 gain headstages (Molecular Devices, Sunnyvale, CA). Data were digitized (model 4/30, PowerLab), acquired and analyzed with Chart 5.5.6 software (AD Instruments software and hardware). On average 5 NMJs were sampled per muscle per experimental condition and at least 5 end-plate potentials (EPPs) and 30 miniature EPPs (mEPPs) were sampled per NMJ. The calculation of quantal content (QC) was done according to McLachlan and Martin [20]. EPP amplitudes were corrected for non-linear summation using the formula: EPP_corrected_  =  EPP/(1 − *f*(EPP/RMP)), where the membrane capacitance factor (*f*) was fixed at 0.8, appropriate for mouse muscle fibers. QC was calculated by dividing mean EPP_corrected_ amplitude by mean mEPP amplitude. Rundown analysis was obtained by recording EPPs at 10 Hz and 40 Hz for 5 seconds and EPP_(n)_ amplitude was quantified using Mini analysis (Synaptosoft, Fort Lee, USA) and expressed as either raw amplitude (mV) or normalized to the initial EPP_(1)_.

Individual recordings from the *Thy1-YFP* ^tg/+^::*Sod1* ^+/+^and *Thy1-YFP* ^tg/+^::*Sod1* ^−/−^ mice were obtained by first identifying each nerve terminal by YFP fluorescence. Images were captured to be used to identify the morphology of the NMJ after fixation and staining with fluorescent conjugated α-bungarotoxin (method described below).

### NMJ imaging

Gastrocnemius and EDL muscles were dissected from *Thy1-YFP* ^tg/+^::*Sod1* ^+/+^ and *Thy1-YFP* ^tg/+^::*Sod1* ^−/−^ mice, pinned on a Sylgard dish and fixed in 4% paraformaldehyde solution for 30 minutes. After several washes with PBS buffer, muscles were incubated with α-bungarotoxin, Alexa Fluor 594 conjugate (2 ug/ml, Molecular Probes, Invitrogen) for 30 minutes to stain post-synaptic acetylcholine receptors. The whole muscles were then washed a few times with PBS, thin filets of muscles were dissected off and mounted with Prolong-Gold mounting media. The innervation quantification was scored manually under an epi-fluorescent microscope following criteria as we described previously [8], [21].

Z-slice images were captured using Olympus FV-1000 Confocal MultiPhoton Spectral Laser Scanning Microscope. Motor axon/nerve terminals and nAChRs were visualized by YFP fluorescence and Alexa Fluor 594 respectively. Image analysis to quantify AChR area of an NMJ and overlap between the axon and AChRs was performed in ImageJ [22]. Briefly, z-maximum intensity projections were constructed from each stack of images. Maximum background intensity was then measured in a ROI devoid of structures and used for applying a threshold. Resultant binary images were used for all calculations. AChR area was calculated as the number of pixels (intensity  =  1), and converted to µm^2^ after calibration. Overlap pixels were obtained by superimposing the axon on AChR binary images. Occupancy values were derived by dividing the number of overlap pixels by AChR pixels for each NMJ. AChR average density estimates (*F_int_/Area*) were produced by calculating the total integrated pixel intensity (*F_int_*) in 16-bit background-corrected average intensity z-projections for α-bungarotoxin-Alexa Fluor 594 staining, and normalizing them to AChR area (*Area*). In some instances, physical positioning of the invading axon and the presynaptic bouton might lead to some degree of overestimation of overlap. Obstructed NMJs were excluded from the analysis.

### Statistical analysis

Electrophysiology data and NMJ morphological parameters are expressed as group mean ± standard error of the mean, and analyzed using paired student *t-test* (two tailed) unless otherwise mentioned. The group mean value was calculated from mean values for individual animals shown as N. The total number of records for all animals is designated as n.

## Results

### Reduced muscle mass and strength in young adult *Sod1^−/−^* mice

**Figure 1:**
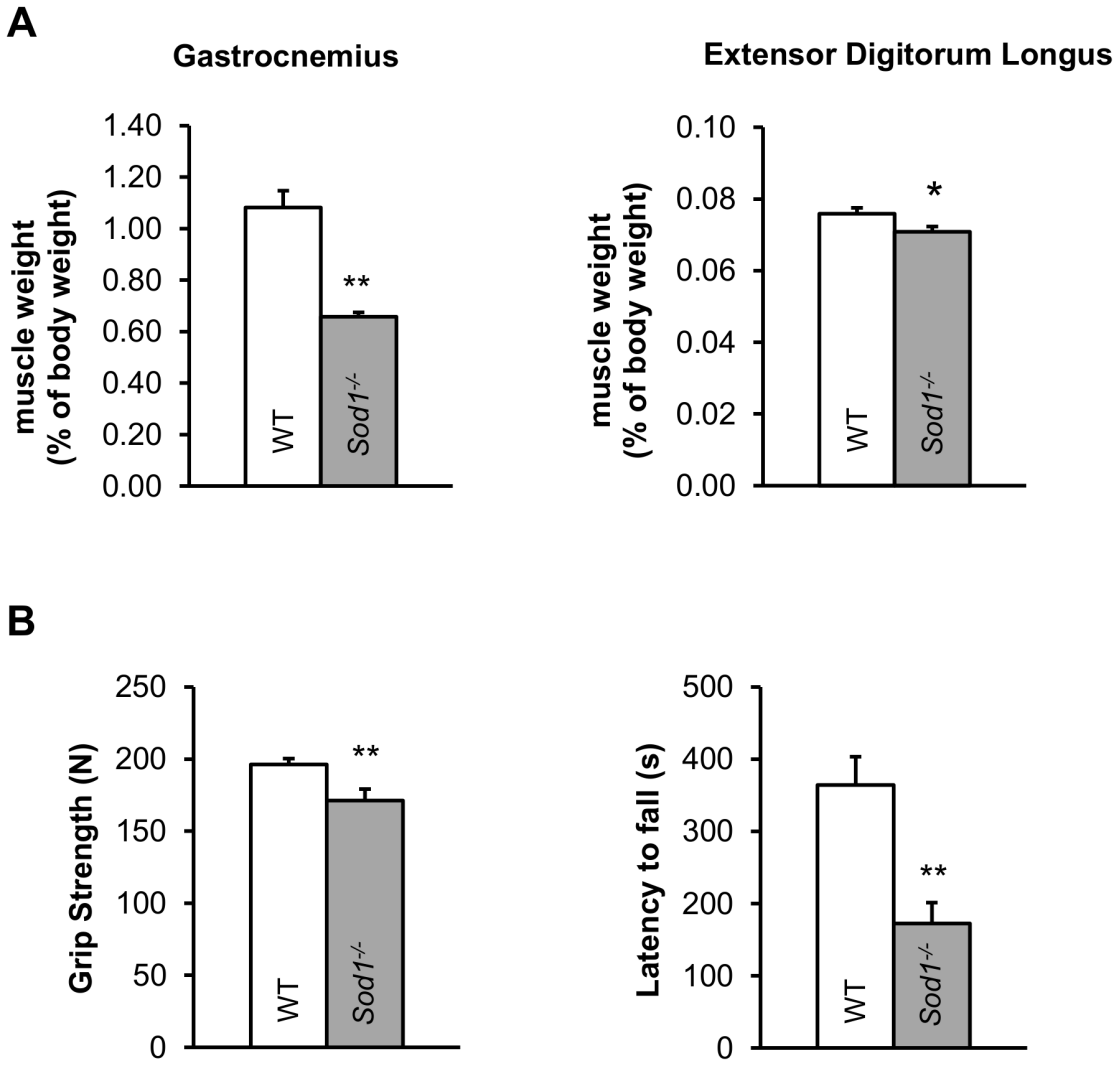
Increased oxidative stress in *Sod1* ^−/−^ mice leads to early onset limb muscle atrophy and weakness. (*A*) Gastrocnemius and extensor digitorum longus muscle mass expressed as % of body mass from in wild-type (WT, N = 47, 7 months old, white) and *Sod1* ^−/−^ (N = 32, 7 months old, gray) mice. (*B, left*) Chart showing the average (10 trials) grip strength in WT (N = 14, 5–6 months old, white) and *Sod1* ^−/−^ (N = 13, 5 months old, gray) mice. (*B, right*) Chart showing time to fall in the wire hanging assay for WT (N = 10, 5–6 months old, white) and *Sod1* ^−/−^ (N = 8, 4–5 months old, gray) mice. All mice are between 4 to 10 months of age. Data are plotted as mean with error bars representing standard error. Statistics: *t-test*, *p<0.05, **p<0.01.

Our previous studies have shown that mice deficient in CuZnSOD (*Sod1* ^−/−^), a model of oxidative stress and sarcopenia, have significantly increased levels of oxidative damage biomarkers, such as F_2_-isoprostanes, protein carbonyls and 8-oxo-dG in various tissues including skeletal muscle [6], [15]. Numerous conditions of muscle wasting and atrophy have been linked to elevated oxidative stress [23]. In agreement with this, *Sod1* ^−/−^ mice also fail to sustain their muscle mass in various hind limb muscles from early adulthood. **Figure 1A** compares muscle mass of the gastrocnemius and extensor digitorum longus (EDL) muscles from young *Sod1* ^−/−^ and wild-type (WT) mice. Both *Sod1* ^−/−^ muscles were smaller than those in WT with the gastrocnemius muscle affected more than the EDL. As anticipated, muscle strength in *Sod1* ^−/−^ mice was also diminished, which is demonstrated by poor performance in grip strength and wire hanging tests (**Fig. 1B**).

### Selective motor nerve/axon, neuromuscular junction pathology in *Sod1^−/−^* mice

**Figure 2:**
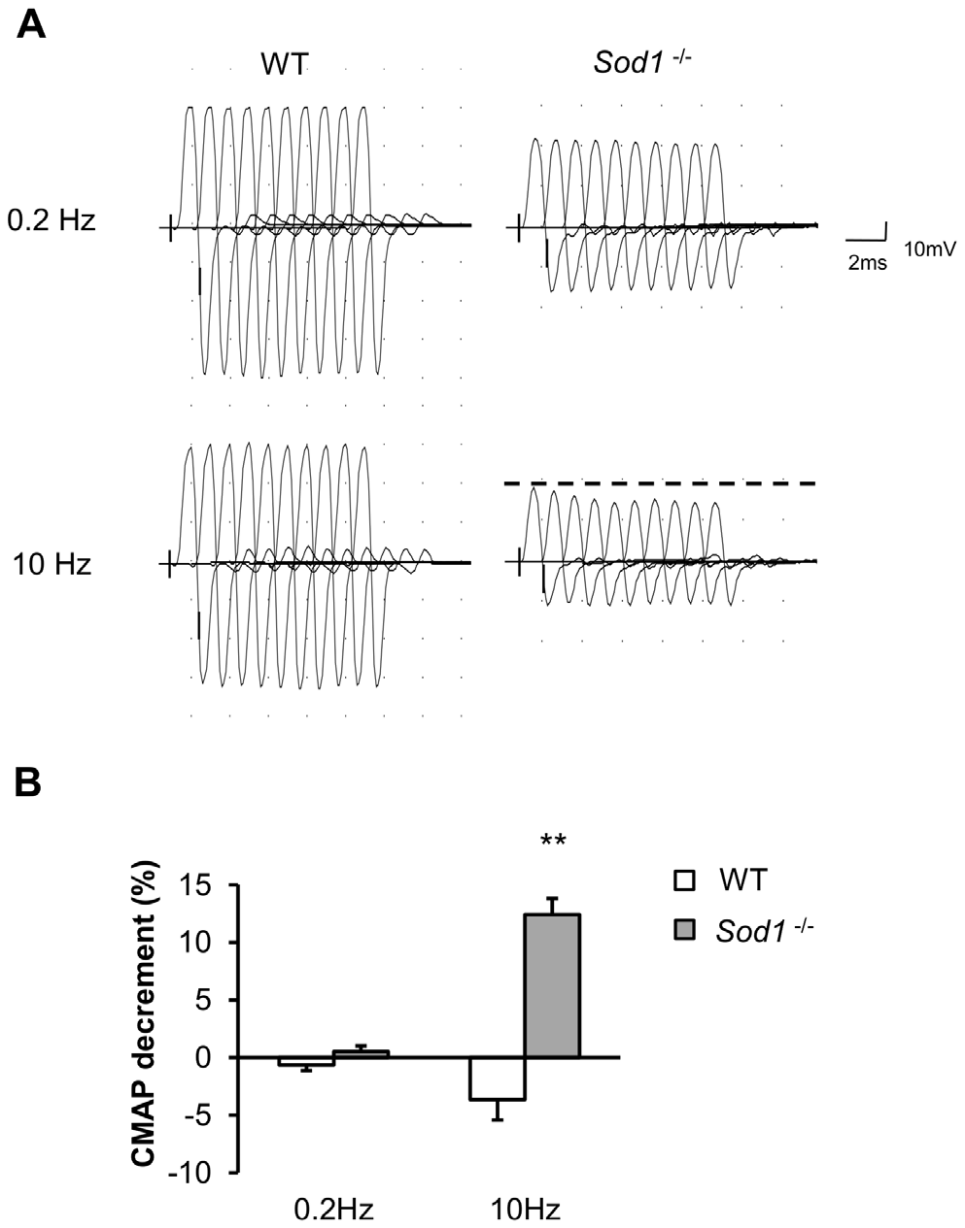
Aberrant electromyographic characteristics in *Sod1* ^−/−^ muscle imply deficits in neurotransmission and denervation. (*A*) Representative traces of gastrocnemius compound muscle action potential (CMAP) when stimulated at 0.2 Hz and 10 Hz. Dashed line shows the amplitude of the initial peak. Traces were arbitrarily spaced 1ms apart for easy visualization. (*B*) Quantitative result of CMAP decrement in WT (N = 17, 7 months old, white) and *Sod1* ^−/−^ (N = 11, 7 months old, gray) mice in response to repetitive nerve stimulation. Data are plotted as mean with error bars representing standard error. All mice are between 4 to 10 months of age. Statistics: *t-test*, *p<0.05, **p<0.01.

To examine the effects of oxidative stress on the peripheral nerve and muscle *in vivo,* we conducted electrophysiological studies in young adult *Sod1* ^−/−^ and WT mice. Nerve conduction measurements were performed to assess the functional status of the peripheral nerve and its ability to conduct an electrical impulse and produce muscle fiber activation. Analysis of the evoked compound muscle action potential (CMAP) elicited by stimulation of the sciatic nerve revealed that both CMAP area and amplitude were diminished in the gastrocnemius muscle in *Sod1* ^−/−^ mice (CMAP amplitude: 24±1 mV, N = 17 versus 36±3 mV, N = 11 for *Sod1* ^−/−^ and WT respectively, p = 0.0014; CMAP area: 25±3 mV*ms N =  17 versus 15±1 mV*ms N = 11 for *Sod1* ^−/−^ and WT, p = 0.0013, paired *t-test*). This decrease in CMAP parameters in *Sod1* ^−/−^ mice could be due to a decrease in the size of muscle fibers, functional muscle fiber denervation and/or inhibition of synaptic release. We also found that there was a significant increase in tail distal motor latency (TDML) and a reduction in sciatic nerve motor nerve conduction velocity (NCV, data not shown). However, sensory nerve conduction velocity (NCV) remained unchanged (in both tail sensory and sural nerve NCV, data not shown). The repetitive nerve stimulation (RNS) test was also performed to determine whether the reduction in muscle contractile force is caused by a deficit in neurotransmission at the NMJ. As expected, in WT mice, there was no reduction in CMAP amplitude when the sciatic nerve was stimulated with a train of ten stimuli at either 0.2 Hz or 10 Hz stimulation frequencies. In contrast, *Sod1* ^−/−^ mice displayed a significant decrement (12%) in CMAP amplitude when stimulated at 10 Hz but no obvious deficit at 0.2 Hz (**Fig. 2A and B**). This might suggest a failure in action potential generation/propagation, which reduces the number of active motor units when the axons are stimulated at higher frequencies. The CMAP decrement was also present at 3 or 5 Hz. Similarly, the tibialis anterior muscle (TA), another muscle innervated by branches of sciatic nerve in *Sod1* ^−/−^ mice, also exhibits comparable reductions in CMAP amplitude and area, and CMAP decrement in RNS test (data not shown). In addition, we performed electromyography tests to assess the spontaneous activities of the muscle without nerve stimulation. Consistent with our predictions of NMJ denervation, we observed increased insertional activity, fibrillation potential and positive sharp waves, which are all characteristics of irregular spontaneous muscle discharge (data not shown). These results indicate that loss of CuZnSOD has a selective effect on motor axons and NMJs relative to the sensory nerve.

### Abnormal spontaneous and evoked acetylcholine release parameters in *Sod1^−/−^* mice

**Figure 3:**
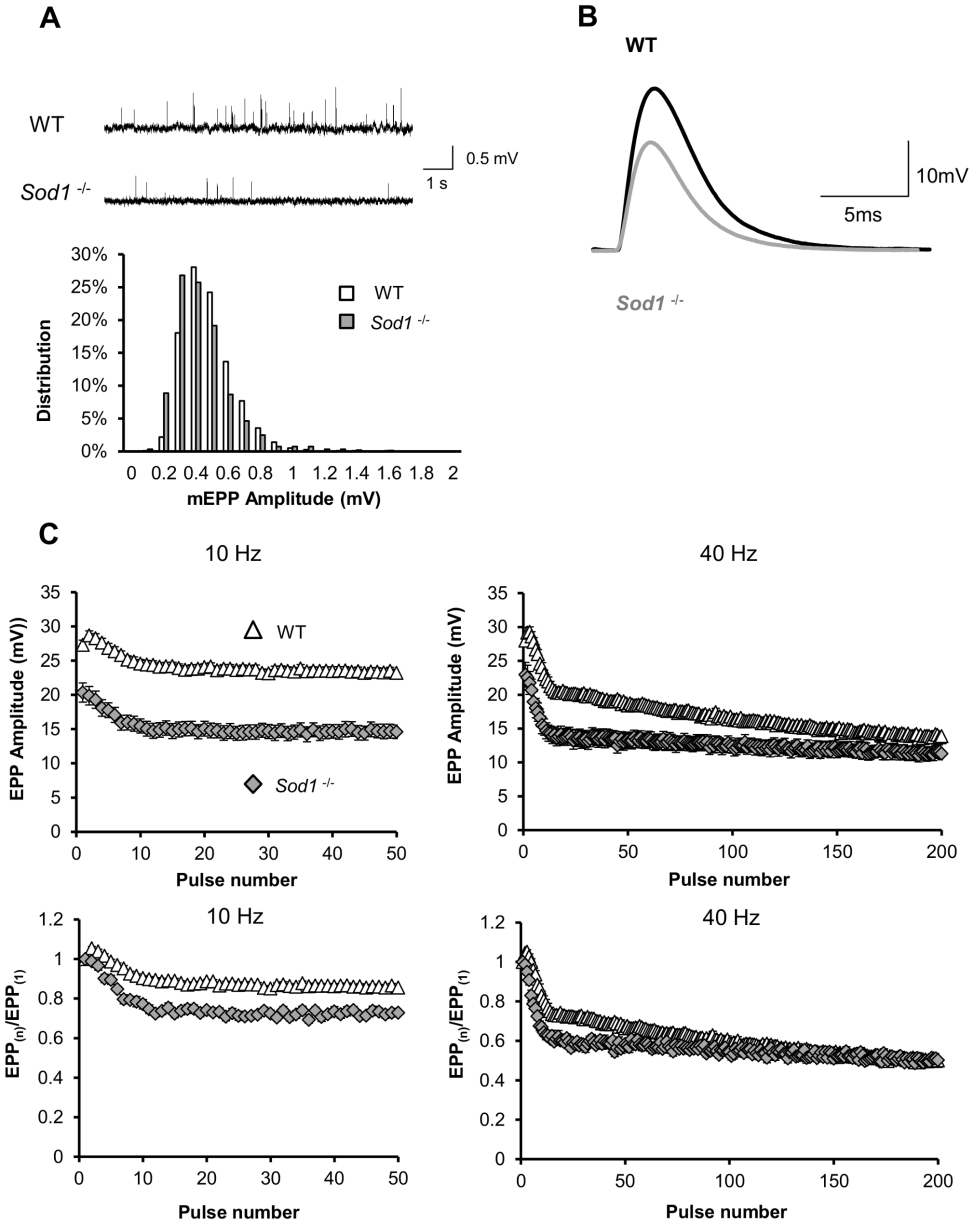
Spontaneous and evoked synaptic release are impaired at *Sod1* ^−/−^ EDL neuromuscular junctions (NMJs). (*A*, *top*) Representative traces of spontaneous mEPPs in wild-type (WT) and *Sod1* ^−/−^ EDL muscles. (*A*, *bottom*) Amplitude-frequency histogram of spontaneous mEPPs for WT (white) and *Sod1* ^−/−^ (gray). Data were analyzed from pooled results from 22 individual records (∼1000 mEPPs) and binned in 0.1 mV intervals. (*B*) Representative traces of evoked EPPs recorded in WT (black) and *Sod1* ^−/−^ (gray) EDL muscle fibers. (*C*) EPP rundown analysis over 5s at 10 Hz and 40 Hz (EPP amplitude, *top*; normalized result, *bottom*) for WT (n = 34∼36 NMJs from N = 5 mice, 5 months old, triangles) and *Sod1* ^−/−^ (n = 22∼28 NMJs from N = 5 mice, 5–6 months old, diamonds).

Nerve conduction assays in *Sod1* ^−/−^ mice showed a reduction in both amplitude and area of CMAP recorded in both gastrocnemius and TA muscles in *Sod1* ^−/−^ mice, which could be the result of changes in synaptic release at individual NMJs. To test this, we studied synaptic transmission in 5-6 month old *Sod1* ^−/−^ and WT mice using the EDL muscle-peroneal nerve preparation due to the similarities in fiber composition with gastrocnemius. On average, EDL muscles from both genotypes had similar resting membrane potential and input resistance values, see **Table 1**. Miniature endplate potential (mEPP) amplitudes remain unaffected, but the frequency of spontaneous mEPPs was nearly five-fold smaller in *Sod1* ^−/−^ mice than in WT mice (**Table 1**, and an example is shown in **Fig. 3A**). At the same time, the average amplitude of stimulus-evoked endplate potentials (EPPs) was significantly smaller in *Sod1* ^−/−^ mice than in WT mice (**Table 1**, and a representative trace is shown in **Fig. 3B**). Quantal content (QC) analysis demonstrates that in EDL muscle from *Sod1* ^−/−^ mice, ∼ 1/3 fewer synaptic vesicles are released per action potential than in EDL muscle from WT mice. This decrease in both quantal content and EPP amplitude in *Sod1* ^−/−^ mice is likely due to a presynaptic deficit, since muscle input resistance, mEPP amplitude and time constant are similar in both groups, and the post-synaptic nAChR density remains unchanged (see below). To further investigate the functional impairment of neurotransmitter release at the NMJ in *Sod1* ^−/−^ mice, we examined sustained release properties by applying 5 s trains of high frequency stimulation. At both 10 Hz and 40 Hz, the rundown of EPP amplitude was faster in *Sod1* ^−/−^ mice than in WT control mice (**Fig. 3C**). Also, in contrast to the immediate increase in EPP amplitude after the first stimulus in WT mice, no paired pulse facilitation was observed in *Sod1* ^−/−^ mice.

**Table 1:** Summary of EDL muscle electrophysiological properties in wild-type (WT) and *Sod1* ^−/−^ mice.

	WT	*Sod1* ^−/−^
**Number of mice (total number of NMJ)**	N = 4 (20)	N = 5 (24)
**Resting membrane potential (mV)**	−73.32±0.53	−71.73±1.43
**Input resistance (MOhm)**	2.71±0.04	2.04±0.31
**mEPP amplitude (mV)**	0.46±0.03	0.44±0.02
**mEPP frequency (Hz)**	3.74±0.59	0.78±0.12**
**t(1/2) of mEPP, (ms)**	2.07±0.06	2.10±0.04
**EPP amplitude (mV)**	27.70±1.61	19.70±1.71*
**Quantal content**	93±8	63±8*

Values are mean ± standard errors. N depicts the number of animals used, and n depicts the total number of NMJs sampled. Statistics: *t-test*, *p<0.05 and **p<0.01.

### NMJ morphological alterations are associated with electrophysiological changes

**Figure 4:**
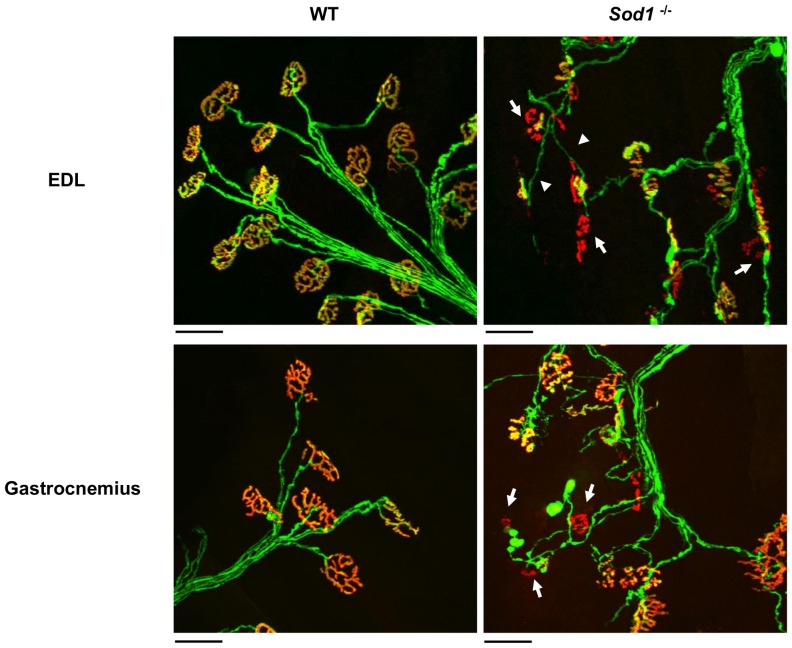
Degenerative morphological alterations in neuromuscular junctions (NMJs) in wild-type and *Sod1* ^−/−^ muscles. NMJs in EDL and gastrocnemius muscles were visualized via Thy1-YFP fluorescence in the presynaptic axon and nerve terminal (green), as described in detail in Materials and Methods, and by staining acetylcholine receptors (nAChRs) with fluorophore conjugated α-bungarotoxin (red). Partially innervated and completely denervated NMJs in *Sod1* ^−/−^ muscle were indicated (arrows). Axon thinning was also frequently observed in *Sod1* ^−/−^ mice (arrow heads). Scale bars: 50µm.

**Figure 5:**
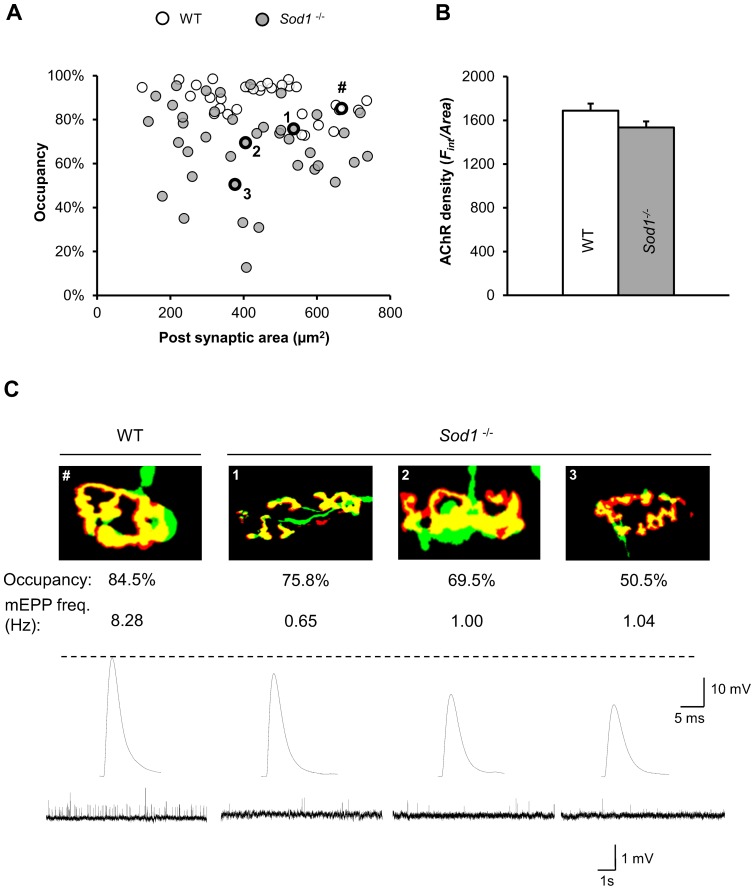
Morphology-synaptic function relationship in wild-type and *Sod1* ^−/−^ EDL neuromuscular junctions (NMJs). (*A*) Scatter plot showing the relationship between AChRs total area and its occupancy by axon shown as % of overlap over the AChRs total area in wild-type (WT, white) and *Sod1* ^−/−^ (gray) mice. NMJs labeled as “#” and “1, 2 and 3” are examples of individual NMJs in WT and *Sod1* ^−/−^ mice which were recorded for electrophysiology shown in (*C*). (*B*) Quantification of average AChR density (*F_int_/Area*) in EDL muscle NMJs from WT (n = 30, white) and *Sod1* ^−/−^ (n = 30, gray) mice. Data are plotted as mean with error bars representing standard error. (*C*) Representative images of individual EDL NMJs showing the overlap (yellow) between axonal (green) and AChRs (red) fluorescence and their respective spontaneous mEPP and evoked EPP electrophysiological responses in WT and *Sod1* ^−/−^ mice. The areas and corresponding occupancies for the NMJs are shown by numbered circles in (*A*).

Changes in the degree of endplate innervation (partial axonal detachment), defined as the degree of overlap between the innervating axon terminal and acetylcholine receptor (AChR) field of a muscle fiber might be responsible for the observed decreases in both EPP amplitude and mEPP frequency in *Sod1* ^−/−^ mice. In order to test this, we first examined the gross morphological alterations in NMJs at the gastrocnemius muscle and EDL muscles in young (4–10 months old) *Sod1* ^−/−^ mice. AChR was labeled using Alexa Fluor 594 conjugated α-bungarotoxin, and presynaptic axon and nerve terminal was visualized with YFP expression (Thy1-YFP). Consistent with previous studies [8], [21], prominent degenerative changes were observed in *Sod1* ^−/−^ muscles, including axon thinning and irregular swelling, complete or partial withdrawal of nerve terminals from the endplate, terminal sprouting, and discontinuous AChRs (**Fig. 4**). Quantification of innervation status was performed under an epi-fluorescent microscope and tabulated in **Table 2**. The imaging results indicate that ∼20% or more of the total endplates in *Sod1* ^−/−^ muscles show some degree of denervation. Furthermore, we used laser scanning confocal microscopy to quantify the degree of AChR-axon overlap and NMJ area (AChRs field area) in fixed and α-bungarotoxin labeled EDL muscles from WT and *Sod1* ^−/−^ mice that express YFP in neurons (*Thy1-YFP* ^tg/+^::*Sod1* ^+/+^ and *Thy1-YFP* ^tg/+^::*Sod1* ^−/−^ mice respectively). Suprisingly, we found that there was no difference in the average NMJ area between *Sod1* ^−/−^ and WT EDL muscles (*Sod1* ^−/−^: 416±27 µm^2^, N = 3, n = 41; WT: 463±26 µm^2^, N = 4, n = 34, p = 0.24; paired *t-test*, **Fig. 5A**). Additionally, the average AChR density (*F_int_/Area*) calculated as integrated fluorescence intensity of Alexa Fluor 594 normalized to the total endplate area was not significantly different between WT and *Sod1* ^−/−^ endplate (**Fig. 5B**). However, the average endplate occupancy by innervating axon was significantly smaller in *Sod1* ^−/−^ than in WT EDL (*Sod1* ^−/−^: 68.8±3.0%, N = 3, n = 41; WT: 89.1±1.3%, N = 4, n = 34; p<0.001, paired *t-test*). Moreover, we recorded both EPPs and mEPPs from individually identified NMJs in *Sod1* ^−/−^ and WT EDL muscles, and also, measured post-fixation their endplate occupancy and area values. In general, smaller occupancies were associated with smaller EPP amplitudes in *Sod1* ^−/−^ mice (**Fig. 5C**). This suggests that the degree of overlap between the axon and AChRs is one of the major factors in determining the size of EPPs and QC in *Sod1* ^−/−^ mice. However, reductions in mEPP frequency were far in excess of that which might have been predicted from the small but significant reduction in endplate occupancy in *Sod1* ^−*/*−^ mice.

**Table 2:** Quantification of neuromuscular junction (NMJ) denervation in wild-type and *Sod1* ^−/−^ muscles.

	Innervated	Partially Innervated	Denervated
***EDL***			
WT (N = 4, n = 809)	100%	0%	0%
Sod1KO (N = 3, n = 928)	80%	16%	4%
***Gastrocnemius***			
WT (N = 3, n = 693)	98%	2%	0%
Sod1 KO (N = 4, n = 736)	68%	23%	8%

N depicts the number of animals used, and n depicts the total number of NMJs sampled.

### Acute administration of 3, 4-diaminopyridine (DAP) improves neurotransmission and muscle performance in *Sod1^−/−^* mice

**Figure 6:**
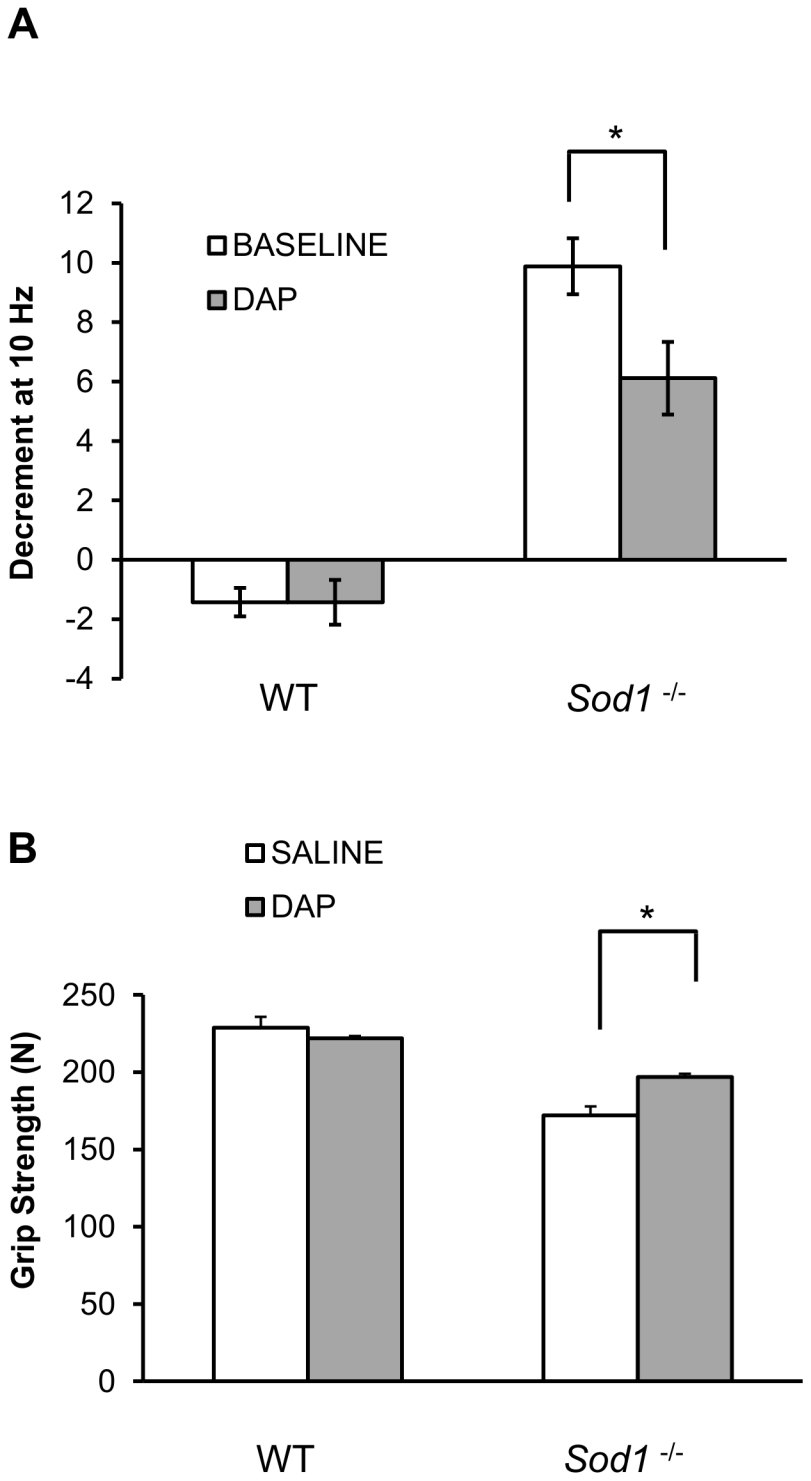
Presynaptic potentiation via potassium channel blocker 3,4-diaminopyridine (DAP) reduces compound muscle action potential (CMAP) deficit and improves grip strength in *Sod1* ^−/−^ mice. (*A*) Decrement of CMAP in response to 10 Hz repetitive stimulation was measured before (white) and 20 minutes after DAP administration (8 mg/kg, i.p., gray) in wild-type (WT, N = 7, 7-8 months old) and *Sod1* ^−/−^ (N = 17, 5–6 months old) mice as described in detail in Materials and Methods. (*B*) Grip strength was tested in wild type (WT, N = 13, 5 months old) and *Sod1* ^−/−^ (N = 11, 5 months old) mice after injection of DAP (1 mg/kg, i.p., gray) or saline (white). Data are plotted as mean with error bars representing standard error. Statistics: *t-test*, *p<0.05, **p<0.01.

RNS tests *in vivo*, and *ex-vivo* electrophysiological recordings from single muscle fibers indicate that the cause of muscle fiber denervation in *Sod1* ^−/−^ mice is likely to be a defect in neurotransmitter release. Muscle fiber denervation is manifest as reduced axonal endplate occupancy and muscle weakness. Such changes might be reversed, and muscle performance might be improved, if neurotransmitter release can be enhanced. We therefore tested the acute effects of 3,4-diaminopyridine (DAP), a non-selective potassium channel blocker that broadens the presynaptic action potential and allows more calcium to enter the terminal. DAP (administered i.p.) significantly diminished the CMAP decrement at 10 Hz in *Sod1* ^−/−^ mice (**Fig. 6A**). It also showed a trend for increased CMAP amplitude in *Sod1* ^−/−^ mice (*p* = 0.09, paired *t-test*, data not shown), whereas it did not alter CMAP assessment of muscle function in WT mice. Moreover, acute administration of DAP augmented grip strength compared to saline treatment in *Sod1* ^−/−^ mice (*p* = 0.02, paired *t-test*), while it did not change grip strength in WT mice (*p* = 0.45, paired *t-test*, **Fig. 6B**). Therefore, pharmacological intervention aimed at increasing neurotransmission can partially improve muscle strength caused by the lack of Sod1.

## Discussion

Oxidative stress is a prominent feature in many muscle wasting conditions. Specifically, increased oxidative stress associated with aging, muscle immobilization, motor neuron degeneration and some autoimmune diseases has been shown to contribute to neuronal and muscle dysfunction, muscle weakness and muscle mass loss [24], [25]. However, it remains unknown whether oxidative stress on its own can modulate neurotransmission and elicit deficits that contribute to muscle dysfunction. To address this question, we evaluated neurotransmission parameters in the CuZnSOD deficient (*Sod1* ^−/−^) mouse, a model of accelerated oxidative stress and sarcopenia that shows muscle atrophy and weakness as early as 5 months of age [6], [26], and in age-matched wild-type (WT) mice. We found a significant reduction in spontaneous, evoked, and sustained neurotransmitter release indicating that synaptic transmission in *Sod1* ^−/−^ hind limb muscles is markedly impaired (**Fig. 3** **and** **5**). In line with the reduction in EPP amplitudes in individual muscle fibers, the amplitudes of compound muscle action potentials (CMAPs) in hind limb muscles of *Sod1* ^−/−^ mice are also significantly smaller than in WT mice (**Fig. 2**), implying that in addition to a decrease in muscle mass (**Fig. 1A** and [7]) there is also a failure in muscle fiber recruitment, which is typical of muscle weakness (**Fig. 1B**). Intraperitoneal injection of 3,4-diaminopyridine (DAP) to *Sod1* ^−/−^ mice, a drug that prolongs action potentials and increases synaptic release, improves CMAP amplitude during repetitive nerve stimulation and increases muscle strength (**Fig. 6**), emphasizing a likely presynaptic deficit as a cause of the muscle weakness. All of these results are in agreement with earlier rescue studies implicating presynaptic dysfunction in *Sod1* ^−/−^ mice [27]–[29] in which re-introduction of CuZnSOD into neurons or only neuronal mitochondria reversed the motor/neuromuscular deficits.

NMJ disassembly is a common pathology seen in aged animals [30]–[32] and also, in a number of degenerative diseases such as amyotrophic lateral sclerosis [32], [33] and spinal muscular atrophy [34]. It is also widely believed that synaptic dysfunction develops concurrently or as a direct result of NMJ structural changes leading to neuropathy and neuronal loss [35]–[38]. However, little is known about the role played by oxidative stress in these NMJ changes. Here we report that *Sod1* ^−/−^ mice have a synaptic release impairment that is accompanied by NMJs degeneration and hind limb muscle weakness and atrophy, supporting our hypothesis of a direct role for oxidative stress in these events. Importantly, our results also suggest that a decrease in synaptic release may in fact precede morphological alterations at the NMJs (**Fig. 4** **and** **Table 2**). Increased ROS production in G93A-SOD1 mice is also accompanied by a marked decline in spontaneous synaptic release in the diaphragm muscle. Granulocyte colony stimulating factor treatment attenuates ROS production in this model and also lessens the presynaptic dysfunction [5].In contrast, majority of NMJs resistant to structural changes in the mouse model of Huntington's disease display huge increases in both mEPP frequency and EPP amplitude [39]. Our findings are also in line with previous studies that utilized different *Sod1* ^−/−^ strains and showed reductions in motor nerve conduction, abnormal NMJ morphologies and muscle fiber grouping [27], [40], indicating that the observed phenotype is not strain specific. Further analysis performed in this study revealed that the reduction in evoked neurotransmitter release correlates with the decrease in axonal occupancy of postsynaptic acetylcholine receptors and muscle weakness (**Fig. 5C**). Most of our data are collected from fast twitch EDL and gastrocnemius muscles, and these ROS dependent declines in muscle and neuronal function are consistent with the greater vulnerability of fast twitch muscles to elevated oxidative stress seen during aging and neurodegeneration [41].

Similar values of mEPP amplitudes, mEPP time constants and muscle input resistances in *Sod1* ^−/−^ and WT mice obtained in this study reinforce our conclusions that the damage to post-synaptic mechanisms follows the damage to the pre-synaptic mechanisms. In support of this, we found that deletion of CuZnSOD restricted to skeletal muscle using a conditional *Sod1* knockout model caused no overt disruptions in synaptic transmission (unpublished data) or morphological denervation [42]. On the other hand, when CuZnSOD is ectopically expressed in neurons in the *Sod1* ^−/−^ mice, NMJ morphology and neurotransmission are fully rescued [29]. Moreover, a study by Fischer et al. (2011) showed that neuronal mitochondrial expression of CuZnSOD in *Sod1* ^−/−^ mice was sufficient to reverse the NMJ degeneration and loss of muscle mass [28]. Together these data strongly support an essential role for the control of superoxide levels in the presynaptic motor neurons by CuZnSOD in NMJ maintenance and function.

The significant reduction in mEPP frequency seen in *Sod1* ^−/−^ mice is independent of NMJ axonal occupancy (**Fig. 5C**). This suggests that NMJs in *Sod1* ^−/−^ mice undergo changes that perturb normal synaptic function before muscle fiber denervation. This could be due to 1) a reduced synaptic vesicle pool size, 2) a decrease in active zone number, or, 3) a change in the probability of release. The unchanged average area and density of AChRs in *Sod1* ^−/−^ mice (**Fig. 5A and 5B**) was unexpected since more fragmented endplates and significantly fewer spontaneous neurotransmitter release events were observed (**Fig. 4** and **Table 2**). The preservation of NMJ size in *Sod1* ^−/−^ mice is associated with the relatively normal muscle fiber size, but reduced fiber number in muscles predominately composed of fast twitch fibers such as the gastrocnemius [7]. However, this does not exclude the possibility that some of these receptors are non-functional or the complex and intricate NMJ assembly is already modified at the ultra-structural level perturbing signaling mechanisms. In fact, a recent report by Chen et al. (2012) has shown a decreased density of the active zone protein Bassoon in aged mouse NMJs that it is independent of partial or full denervation [43]. Therefore it is possible that active zone density is diminished even in the still innervated *Sod1* ^−/−^ NMJs. Neurotransmitter synthesis and recycling are energy consuming processes [44], and a ROS dependent impairment in mitochondrial function, particularly in ATP synthesis, could cause deficits in synaptic vesicle dynamics and exocytosis [45], [46]. For instance, diminished levels of the readily release pool of synaptic vesicles were reported in diaphragm muscles of SOD1-G93A mice which is associated with increased ROS production [5]. Previous studies from our group have shown that mitochondria dysfunction and reduced ATP production occurs in skeletal muscle as a result of Sod1 deletion [6], [8], [21]. Hence it is possible that loss of CuZnSOD in nerve terminals might lead to a drop in vesicle pool size and a decrease in neurotransmitter release. Finally, voltage dependent calcium channels and synaptic vesicle proteins are sensitive to oxidant inactivation [47], [48]. Thus, excessive ROS production by dysfunctional mitochondria in the absence of CuZnSOD may result in oxidative modifications to susceptible molecules, impacting synaptic transmission and the integrity of the NMJs.

Reduction in muscle mass in young adult (4–10 months) *Sod1* ^−/−^ mice (**Fig. 1A**) and diminished muscle strength (**Fig. 1B**). In particular, *Sod1* ^−/−^ mice showed a more pronounced deficit in wire hanging test than the grip strength assay (**Fig. 1B**). It is probably due to a continuous muscle contraction, thus a sustained presynaptic input the wire hanging test requires. Muscle atrophy and weakness in *Sod1* ^−/−^ mice is consistent with previous reports [6]–[8]. Specially Larkin et al. (2011) examined these animals as early as 1 month of age and found no overt change in muscle mass or strength [7]. This suggests that *Sod1* ^−/−^ mice and WT mice are equivalent in neuromuscular properties early in life. The phenotypes of smaller muscle mass and muscle weakness due to insufficient neurotransmission in *Sod1* ^−/−^ mice likely ensue sometime between 1 month and 5 months. This implies that prolonged exposure to ROS due to the lack of CuZnSOD leads to neuromuscular dysfunction and ultimately results in muscle weakness and atrophy.

Changes in synaptic transmission at NMJs are typically associated with increased muscle fatigability and weakness, and are evident in such disorders as myasthenia gravis (MG) and Lambert-Eaton syndrome (LES). Both of these syndromes involve autoimmune reactions against NMJ components which lead to a marked impairment in sustained neurotransmission and inhibition of muscle action potential initiation. DAP, a potassium channel blocker has been successfully used in several clinical trials in LES patients resulting in some improvements in muscle strength. Experimentally, it has also been shown to improve neurotransmission in a MuSK antibody-induced mouse model of MG both acutely and chronically [49], [50]. DAP administration also increases muscle force, ameliorates fatigue and reduces the neurotransmission failure component of fatigue in rat diaphragm muscles [51]. In this study, reduced synaptic release seen in *Sod1* ^−/−^ mice was mitigated by an acute administration of DAP (**Fig. 6A**). In addition, muscle strength was enhanced (**Fig. 6B**). DAP is a nonspecific potassium channel inhibitor, it can indeed affect both pre- and post-synaptic potassium channels [51] by prolonging the action potential repolarization phase. Different types of muscle fibers and even muscle fiber states such as partially or completely denervated are also associated with different sensitivities to DAP [52]. Therefore, it is very hard to predict if DAP would produce any inhibitory effect in the muscle without directly measuring force/ionic currents in that specific muscle. In this study, there is only a slight DAP effect on muscle strength in wild-type compared to *Sod1* ^−/−^ mice, suggesting it affect neurotransmission but not completely ruling out changes in EC coupling. Nonetheless, the reduction in CMAP decrement should be attributed to its presynaptic effect since CMAP decrement is reflective of a change in the number of activated muscle fibers. Taken together, these findings support the hypothesis of the functional impairment of synaptic transmission as a primary factor that underlies muscle weakness and muscle atrophy in *Sod1* ^−/−^ mice.

In conclusion, we demonstrated that at the synaptic level there is profound functional impairment in neurotransmission in young adult *Sod1* ^−/−^ mice which may contribute to reduced muscle strength and loss of muscle mass. Oxidative stress due to the lack of CuZnSOD specifically in the presynaptic neurons and nerve terminals likely gives rise to the abnormal neurotransmitter release. A better understanding of the interplay between the NMJ functional parameters and NMJ morphological maintenance in genetic mouse models will lead to further insights into synaptic plasticity in diseases and aging.
